# Lesson of the month: novel method to quantify neutrophil uptake in early lung cancer using SPECT-CT

**DOI:** 10.1136/thoraxjnl-2020-214642

**Published:** 2020-09-04

**Authors:** Neda Farahi, Daniel Gillett, Mark Southwood, Doris Rassl, Nicola Tregay, Uta Hill, Stephen Denis Preston, Chrystalla Loutsios, Laurence Si Chong Lok, Sarah Heard, John Buscombe, Robert Campbell Rintoul, A Michael Peters, Charlotte Summers, Edwin R Chilvers

**Affiliations:** 1 Department of Medicine, University of Cambridge, Cambridge, UK; 2 Department of Nuclear Medicine, Cambridge University Hospitals NHS Foundation Trust, Cambridge, UK; 3 Department of Pathology, Royal Papworth Hospital NHS Foundation Trust, Cambridge, UK; 4 Cambridge Centre for Lung Infection, Royal Papworth Hospital NHS Foundation Trust, Cambridge, UK; 5 Department of Thoracic Oncology, Royal Papworth Hospital NHS Foundation Trust, Cambridge, UK; 6 Department of Oncology, University of Cambridge, Cambridge, UK; 7 Department of Nuclear Medicine, King's College Hospital NHS Foundation Trust, London, UK; 8 National Heart and Lung Institute, Imperial College, London, UK

**Keywords:** lung cancer, neutrophil biology, imaging/CT MRI etc

## Abstract

Neutrophils play an important role in the lung tumour microenvironment. We hypothesised that radiolabelled neutrophils coupled to single-photon emission CT (SPECT) may non-invasively quantify neutrophil uptake in tumours from patients with non-small cell lung cancer. We demonstrated increased uptake of radiolabelled neutrophils from the blood into tumours compared with non-specific uptake using radiolabelled transferrin. Moreover, indium-111-neutrophil activity in the tumour biopsies also correlated with myeloperoxidase (MPO)-positive neutrophils. Our data support the utility of imaging with In-111-labelled neutrophils and SPECT-CT to quantify neutrophil uptake in lung cancer.

## Introduction

Lung cancer is the leading cause of cancer-related death worldwide. Inflammation has emerged as a hallmark of tumourigenesis, contributing to the development and progression of a range of cancers. Neutrophils are known to be important players in the tumour microenvironment and these tumour-associated neutrophils (TANs) are broadly classified into an N1 tumour-suppressing phenotype or an N2 tumour-promoting phenotype. While neutrophils may exert both pro-tumorous and antitumorous functions, the prevailing view is that they serve to exclude T cell access and hence promote tumour progression. In murine studies, Ly-6G-specific depletion of neutrophils reduced the metastatic burden in the lungs,^1^ while in *kras*-driven mouse models of lung cancer, Gr1^+^ neutrophils were found to favour tumour growth, reduce T cell homing, and antagonise anti-programmed cell death protein 1 (PD-1) immunotherapy.^2^ Furthermore, neutrophil derived mediators have been shown to be pro-tumourigenic; neutrophil elastase contributes to tumour proliferation,^3^ MMP-9 increases vascular endothelial growth factor release (from the extracellular matrix) to promote angiogenesis,^4^ and neutrophil extracellular traps can sequester circulating tumour cells in the pulmonary microvasculature, promoting the development of metastasis.^5^

Although evidence exists that infiltrating neutrophils accumulate in non-small cell lung cancer (NSCLC),^6^ direct non-invasive imaging and quantification of neutrophils within patient tumours has never been described. Such quantification could aid in predicting the therapeutic response and clinical outcome of patients. Here we describe quantitative single-photon emission CT (SPECT)-CT using indium-111-radiolabelled autologous neutrophils to quantify neutrophil uptake in tumours of patients with early stage lung cancer, using indium-labelled transferrin as a marker for non-specific uptake of non-cell-bound activity. We also correlated tissue concentrations of radiolabelled neutrophils with histological evidence of MPO^+^ neutrophils. These findings have important implications for the use of quantitative SPECT-CT to monitor the extent of neutrophil uptake by tumours.

## Clinical cases

Six patients were selected for entry into the study met the following criteria: (1) positron emission tomography (PET) or biopsy confirmed preoperative stage I–II lung cancer in the mid-upper zones of the lung, (2) no prior chemotherapy or radiotherapy and (3) no other active malignancy. Biopsies were performed ≥1 month prior to acquisition of SPECT images. Tumours in the mid-upper zones were chosen to avoid potential overlap of the signal with physiological uptake of radiolabelled cells in the liver. Patient characteristics can be found in online supplementary table 1 and the study protocol is summarised in online supplementary figure 1.

10.1136/thoraxjnl-2020-214642.supp1 Supplementary data

10.1136/thoraxjnl-2020-214642.supp2 Supplementary data

In four volunteers, neutrophils were isolated from peripheral venous blood using plasma-Percoll gradients and radiolabelled with indium-111-tropolonate, as described in online supplementary files. Given that increased vascularity of tumours could contribute to enhanced uptake of In-111-neutrophils,^7^ we also assessed the uptake of In-111-transferrin into tumours. Transferrin can be taken up by tumours non-specifically through highly permeable blood vessels. Hence, in two further patients, In-111-transferrin was prepared as a control for non-specific uptake (online supplementary files), so that specific focal accumulation of In-111-neutrophils in the tumour area can be compared.

Over 40 min of dynamic gamma camera imaging, labelled neutrophils displayed physiological kinetics in liver, lungs and spleen, in line with previous results from healthy volunteers (online supplementary figure 2A).^8^ The recoveries of radiolabelled neutrophils from the peripheral blood 45 min after injection (online supplementary figure 2B) were comparable to published data, indicating that the cells were not activated by isolation and radiolabelling. As expected, In-111-transferrin behaved as a labelled plasma protein (online supplementary figure 2A, B).

10.1136/thoraxjnl-2020-214642.supp3 Supplementary data

**Figure 1:**
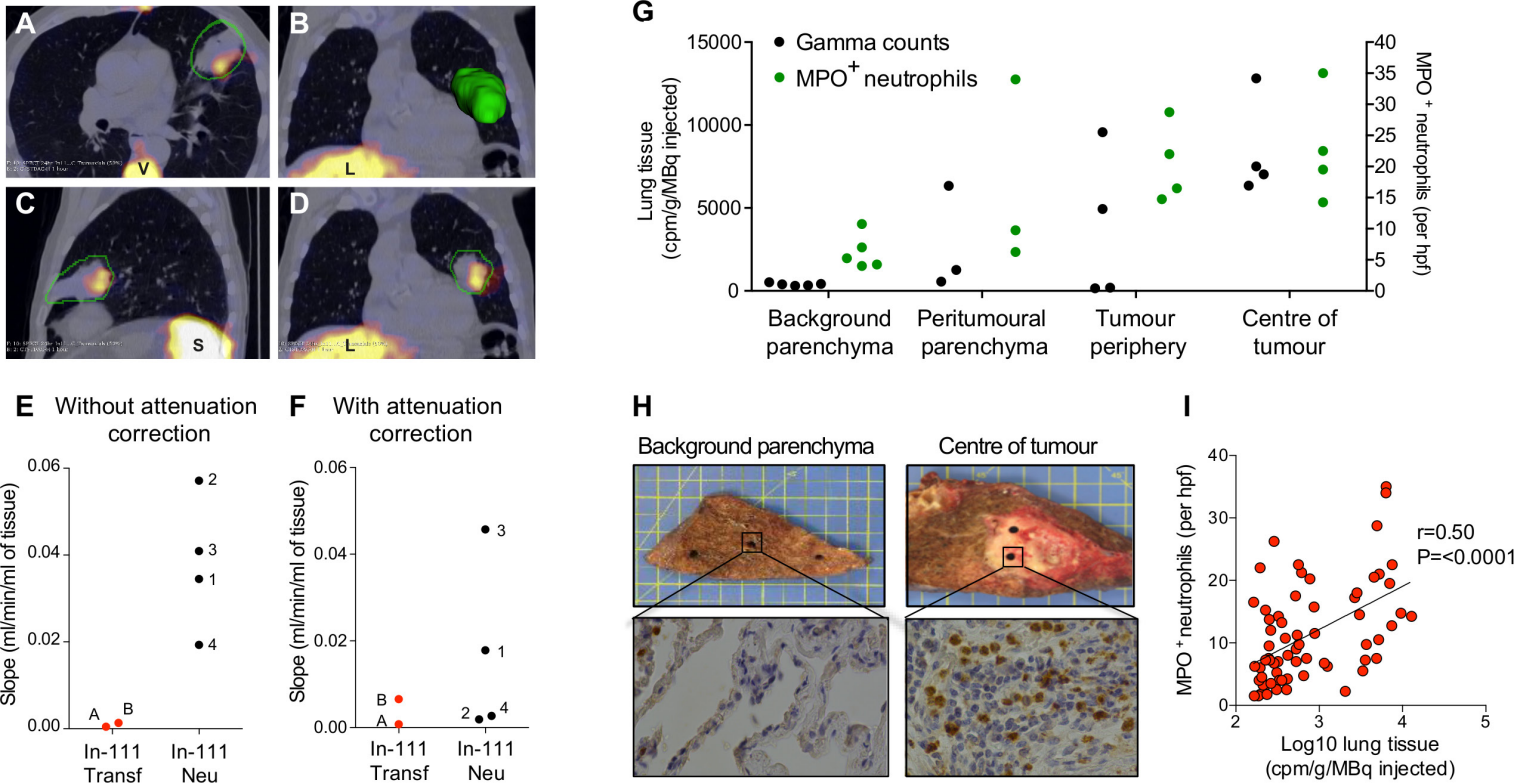
Quantification of radiolabelled neutrophils and radiolabelled transferrin in lung tumours of patients with early stage lung cancer. SPECT-CT images showing (A) transaxial, (B) coronal with three-dimensional reconstruction of tumour, (C) sagittal and (D) coronal plane from a patient with squamous cell carcinoma (case 1). Images show accumulation in the tumour (outlined in green) and physiological uptake in the liver (L), spleen (S) and vertebra (V) 20 hours after reinjection of In-111-labelled neutrophils. (E) and (F) Quantification of tumour uptake of In-111-labelled neutrophils (cases 1–4) and In-111-labelled transferrin (cases A and B) using non-attenuation corrected (E) and attenuation corrected values (F). (G) Tissue gamma counts and MPO^+^ neutrophil counts from the background parenchyma, peri-tumorous parenchyma, tumour periphery and tumour centre of a subject with squamous cell carcinoma (case 1). (H) Background parenchyma and tumour centre cores from case 1 (upper panels) alongside representative myeloperoxidase (MPO) immunohistochemical staining (lower panels). Magnification ×400. (I) Correlation between gamma radiation counts and MPO^+^ neutrophil counts in four subjects. MPO^+^ neutrophils were counted in four high powered fields. Correlation coefficients were calculated using Spearman correlation analysis.

SPECT-CT showed very clear and evident uptake of neutrophils into the tumour of a patient with squamous cell carcinoma (case 1; figure 1A–D). As shown in figure 1E, tumour uptake increased in patients with injected In-111-neutrophils with a median of 0.0038 mL/min/mL (IQR; 0.0231–0.0531) compared with a median of 0.0009 mL/min/mL for In-111-transferrin (IQR; 0.0004–0.0013). SPECT-CT with attenuation correction (figure 1F) showed an overall increase in In-111-neutrophil clearance into tumour with a median of 0.0103 mL/min/mL (IQR; 0.0021–0.0387) compared with a median of 0.0037 mL/min/mL for In-111-transferrin clearance (IQR; 0.0008–0.0066). Of note, SPECT-CT without attenuation correction increased In-111-neutrophil clearance compared with In-111-transferrin clearance in all four patients, but only in two patients following attenuation correction. Attenuation correction of SPECT images is known to improve quantitative accuracy of malignant lesions in the body by reducing soft tissue artefacts.^9^ Therefore, we propose that the attenuation corrected values provide a more representative quantification of neutrophil clearance.

The median peripheral neutrophil count was higher in the In-111 neutrophil patient group compared with the In-111 transferrin group (online supplementary table 1); however, the correlation coefficient between the neutrophil count and the non-attenuation corrected Patlak-Rutland slope (r=0.6, p=0.24) or the attenuation corrected Patlak-Rutland slope (r=0.20, p=0.71) was not significant for the six cases.

On cell counting of tissue ex vivo, case 1 displayed substantial accumulation of neutrophils in the centre (median 21 MPO^+^ neutrophils per hpf (IQR; 14–35) and periphery (median 19 MPO^+^ neutrophils per hpf (IQR; 15–29) of the tumour compared with the background parenchyma (median 5 MPO^+^ neutrophils per hpf (IQR; 4–11) (figure 1G, H). Similar findings were obtained in one further patient with a squamous cell carcinoma (online supplementary fig 3). There was no discernible neutrophil infiltration at the tumour centre or periphery in the patient with adenocarcinoma (online supplementary fig 3). When all four cases were combined, the radiolabelled neutrophil gamma counts positively correlated with the number of MPO^+^ neutrophils (figure 1I; r=0.50, 95% CI 0.291 to 0.666; p<0.0001). Of interest, neutrophils were detected by immunohistochemistry (IHC) when the gamma radiation counts were low, suggesting the presence of tissue resident neutrophils.

10.1136/thoraxjnl-2020-214642.supp4 Supplementary data

## Discussion

In the present study, we demonstrated quantitative in vivo SPECT-CT imaging of radiolabelled neutrophil uptake in tumours of patients with lung cancer. Our study also established that MPO^+^ neutrophils in lung resections correlated with radiolabelled neutrophil counts. Such a non-invasive imaging strategy could have important implications for monitoring neutrophil infiltration into tumours, and assessing the impact of neutrophil-targeted therapies in NSCLC.

Two previous studies have reported enhanced uptake of In-111 mixed leukocytes and In-111 granulocytes into a number of localised malignant neoplasms such as pulmonary sarcoma, although without quantitative measurements and a subjective scoring protocol.^10 11^ There is current interest in quantifying tissue concentration of single-photon emitting tracers using attenuation-corrected SPECT-CT, with the aim of bringing SPECT-CT in line with PET-CT; we believe ours is the first attempt to do so in cell trafficking studies. The importance of our approach lies in the ability to fully exploit techniques such as Patlak-Rutland analysis, which, as in PET-CT, rely on absolute tissue quantification. In our previous studies of neutrophil trafficking in lung tissue, we circumvented this by normalising the Patlak-Rutland slope to the intercept of the plot, which reflects distribution volume of tracer in the tissue (the marginated pulmonary neutrophil pool). However, in solid tissue such as tumour, the quantitative nature of the intercept is uncertain. Quantitative SPECT-CT has general value in studies based on labelled neutrophils because quantification of neutrophil accumulation in enclosed spaces, like abscesses (as opposed to open tracts), uses crude indices such as abscess/background ratios at arbitrarily selected time points post-injection.

Of interest, we observed inter-tumour heterogeneity with respect to neutrophil uptake into tumours by both imaging and immunohistochemistry approaches, consistent with previous immunohistochemistry studies. A larger scale study will be required to understand the prognostic implication of such variable neutrophil infiltration. Some authors have suggested that, in addition to neutrophil abundance, the specific location of neutrophils within the tumour has prognostic relevance. Sody *et al*, using a murine model of neutrophil trafficking, found that neutrophils exhibit distinct migratory patterns depending on their localisation to intra-tumorous or peri-tumorous regions.^12^

Given the broadly pro-tumourigenic role of neutrophils, modulation of neutrophil migration into tumours is an attractive therapeutic approach in NSCLC. Indeed, targeting neutrophil recruitment may be beneficial as adjuvant therapy. The CXCR2 pathway has a major role in the control of neutrophil recruitment into tumours, and antagonists of the CXCR2 receptor have reduced tumour growth in murine studies. Phase II clinical trials using CXCR2 inhibitors in NSCLC patients are on-going.^13^ Our findings thus offer the prospect of imaging neutrophils in clinical trials to better understand the therapeutic response of targeting neutrophils.

Our study has several limitations. First, our results are drawn from a small sample size, and follow-up will be required to confirm the utility of neutrophil imaging in NSCLC. Second, as TANs exhibit significant heterogeneity, our study could not detect the maturity or activation status of the trafficked cells. Third, there are reports of increased transferrin receptor expression on lung cancer cells,^14^ which could limit the use of radiolabelled transferrin as a negative control. However, as both radiolabelled transferrin and radiolabelled neutrophils are injected in plasma, this will likely increase the baseline uptake in both cases.

In conclusion, we demonstrate how radiolabelled neutrophils and SPECT-CT can be combined to allow non-invasive quantification of neutrophil accumulation in lung cancer. Further studies of in vivo neutrophil trafficking in NSCLC are warranted.
