# Supplementary material for: Lesson of the month: novel method to quantify neutrophil uptake in early lung cancer using SPECT-CT
Source: Thorax. 2020 Sep 4;75(11):1020–3. doi: 10.1136/thoraxjnl-2020-214642 (PMC7569370; doi:10.1136/thoraxjnl-2020-214642)
Supplement: Supplementary data [file thoraxjnl-2020-214642supp001.pdf]

## Supplementary Methods, Tables and Figures

### Neutrophil and transferrin radiolabelling

In four volunteers, neutrophils were isolated from peripheral venous blood using plasma-Percoll gradients (GE Healthcare, Buckinghamshire, United Kingdom) and radiolabelled with indium-111-tropolone, as described.[1] Briefly, 80 mL of blood was collected in 5% acid citrate dextrose before erythrocyte sedimentation using 5% hydroxyethylstarch (Grifols). After sedimentation, the upper layer of leukocytes was resusupended in 2 mL cell-free plasma before over layering onto Percoll iso-osmotic gradients (50%, 60%, and 65%) diluted in cell-free plasma. After centrifugation for 5 min at 150g the neutrophil layer was removed from the lower gradient interface before resuspension in cell free plasma and incubation with In-111 (Covidien Healthcare, Fareham, UK) and tropolone (0.054% w/v; Ipswich Pharmacy Manufacturing Unit, Ipswich UK) for 10 min. Prior to radiolabelling, a sample of the neutrophil layer was cytocentrifuged to assess the purity of the isolated fraction (≥95% neutrophil purity, with eosinophils and monocytes being the main contaminating cell types). The recovery of In-111-neutrophils from the peripheral blood was measured 45 min after injection.[2]

In two further patients, In-111-transferrin was prepared as a control for non-specific uptake from 40 mL of autologous venous blood, as described.[3]

### Planar dynamic and SPECT-CT imaging

Participants were positioned in a double-headed SPECT-CT camera (GE Discovery 670, GE Healthcare), fitted with medium-energy, parallel-hole collimators. After bolus injection of indium-111-labelled neutrophils (median 12.9; interquartile range 10.4-14.8 MBq) or indium-



Farahi N, *et al. Thorax* 2020; 75:1020–1023. doi: 10.1136/thoraxjnl-2020-214642

111-labelled transferrin (median 12.1; interquartile range 9.6-14.5 MBq) into an antecubital fossa vein, activities in the chest and upper abdomen were recorded over 40 min.[4] SPECT images were acquired at 1, 4 and 20 h following injection. A single low-dose CT was performed at 45 min. Supplementary Fig.1 summarises the study design.

**Patlak-Rutland analysis**

Patlak-Rutland analysis allows the quantification of the clearance of a tracer from the blood compartment into the tissue. It plots the ratio of tissue-to-blood concentration at known time points after tracer injection against normalised time, which is a derivative of blood concentration. The slope of the plot represents tracer clearance.

Peripheral blood samples were taken at 2, 4, 6, 8, 10, 15, 30, 45, 90, 120, 240, 360, and 1200 min after injection. Regions of interest were drawn on SPECT-CT images. As previously described for whole lung neutrophil clearance, Patlak-Rutland was used to measure In-111-neutrophil and In-111-transferrin clearances from the blood into tumours.[4] For the first time in relation to labelled blood cell trafficking, attenuation correction was performed using Volumetrix (Version MI, GE Healthcare) and SPECT-CT co-registration using 3D Slicer (Version 4.10.1, NIH). Co-registration of the SPECT images before reconstruction used iterative reconstruction (OSEM 2 iterations 10 subsets). Clearances were then expressed without and with attenuation correction, the latter giving clearance in real units of mL/min/mL.

**Tissue quantification of radiolabelled neutrophils**

Punch biopsy cores (4 mm) were taken from formalin-fixed resected tumours ≤24 h after surgery. Cores were taken from the background parenchyma, peri-tumoural parenchyma,



Farahi N, *et al. Thorax* 2020; 75:1020–1023. doi: 10.1136/thoraxjnl-2020-214642

tumour periphery and tumour centre. The number of cores collected from each case is shown in Supplementary Table 2; core number was dependent on lobe size. Furthermore, care was taken to ensure enough samples were available for tumour, node and staging classification. After collection the cores were measured in a gamma counter (Wallac 1480, Perkin Elmer, MA, USA). Radioactivity counts were adjusted for weight, decay-corrected, and expressed as counts/g tissue/MBq.

**Immunohistochemistry**

Four micrometer tissue sections were incubated with polyclonal rabbit anti-human myeloperoxidase antibody (Dako Cytomation, Ely, UK), labelled using dextran-coupled peroxidase and 3-3’diaminobenzidine (Dako Cytomation, Ely, UK), and counterstained with haematoxylin. $MPO^+$ neutrophils were counted in a blinded manner.

**Statistical analysis**

Statistical analyses were undertaken using GraphPad Prism (6.0d, San Diego, CA, USA).

**Table S1. Patients and tumour characteristics.**

Data are presented as median with the interquartile range in brackets. Tumour, node and metastasis staging was classified using The Eighth Edition Lung Cancer Stage Classification.[5]

| | **Indium-111-labelled neutrophils** | **Indium-111-labelled transferrin** |
|---|---|---|
| N | 4 | 2 |
| Age (y) | 72 (69-74) | 75 (71-79) |
| Sex: male/female | 3/1 | 1/1 |
| Peripheral blood neutrophil count (x$10^9$/L) | 6.07 (4.48-7.86) | 3.49 (2.76-4.22) |
| Smoking status (current/former/never) | 0/4/0 | 0/1/1 |
| Cancer type | Lepidic adenocarcinoma (1)<br>Squamous cell carcinoma (3) | Adenocarcinoma (2) |
| Tumour stage | Stage Ib (1)<br>Stage IIb (2)<br>Stage IIIa (1) | Stage IIa (1)<br>Stage IIb (1) |
| Tumour grade | T2a (2), T2b (2) | T2b (2) |
| Nodal stage | N0 (3), N2 (1) | N0 (2) |



Farahi N, *et al. Thorax* 2020; 75:1020–1023. doi: 10.1136/thoraxjnl-2020-214642

**Table S2. Number and location of lung tissue cores collected per clinical case.**

| Case ID | Background parenchyma | Peri-tumoural parenchyma | Tumour periphery | Tumour centre | Total |
|---|---|---|---|---|---|
| 1 | 5 | 3 | 4 | 4 | 17 |
| 2 | 2 | 2 | 12 | 7 | 23 |
| 3 | 4 | 3 | 4 | 2 | 13 |
| 4 | 4 | 3 | 3 | 5 | 15 |

**References**

1 Danpure HJ, Osman S. Optimum conditions for radiolabelling human granulocytes and mixed leucocytes with 111In-tropolonate. *Eur J Nucl Med* 1988;**13**:537–42.

2 Lok LSC, Farahi N, Juss JK, *et al.* Effects of tocilizumab on neutrophil function and kinetics. *Eur J Clin Invest* 2017;**47**. doi:10.1111/eci.12799

3 Carpani De Kaski M, Peters AM, Bradley D, *et al.* Detection and quantification of protein-losing enteropathy with indium-111 transferrin. *Eur J Nucl Med* 1996;**23**:530–3. doi:10.1007/BF00833387

4 Tregay N, Begg M, Cahn A, *et al.* Use of autologous (99m)Technetium-labelled neutrophils to quantify lung neutrophil clearance in COPD. *Thorax* Published Online First: January 2019. doi:10.1136/thoraxjnl-2018-212509

5 Detterbeck FC, Boffa DJ, Kim AW, *et al.* The Eighth Edition Lung Cancer Stage Classification. *Chest* 2017;**151**:193–203. doi:10.1016/j.chest.2016.10.010

**Supplemenray Figure legends**

**Supplementary Figure 1. Summary of study protocol.**

Schematic diagram of the study protocol. Four patients were imaged using In-111 radio-labelled neutrophils and two patients with In-111 radio-labelled transferrin. Lobectomy was performed ≥24 hours following the final SPECT/planar scan. SPECT, single-photon emission computed tomography.

**Supplementary Figure 2. Dynamic planar imaging in patients with resectable lung cancer.**

A) Distribution of radioactivity over 20 h for the right lung (green), left lung (orange), liver (pink), spleen (red) and right ventricle (blue) after reinjection of In-111-labelled neutrophils (left) or In-111-labelled transferrin (right). Data show median and interquartile range. B) Proportion of In-111-labelled neutrophils (n=4) and In-111-labelled transferrin (n=2) remaining in the blood 45 minutes after reinjection.

**Supplementary Figure 3. In-111-labelled neutrophil radioactivity and MPO$^+$ neutrophil counts in resected lungs.**

Tissue gamma counts and MPO$^+$ neutrophil counts from the background parenchyma, peritumoural parenchyma, tumour periphery, and tumour centre from Patients 2, 3, and 4.



Farahi N, *et al. Thorax* 2020; 75:1020–1023. doi: 10.1136/thoraxjnl-2020-214642
