# Supplementary figures and images for: Lesson of the month: novel method to quantify neutrophil uptake in early lung cancer using SPECT-CT
Source: Thorax. 2020 Sep 4;75(11):1020–3. doi: 10.1136/thoraxjnl-2020-214642 (PMC7569370; doi:10.1136/thoraxjnl-2020-214642)

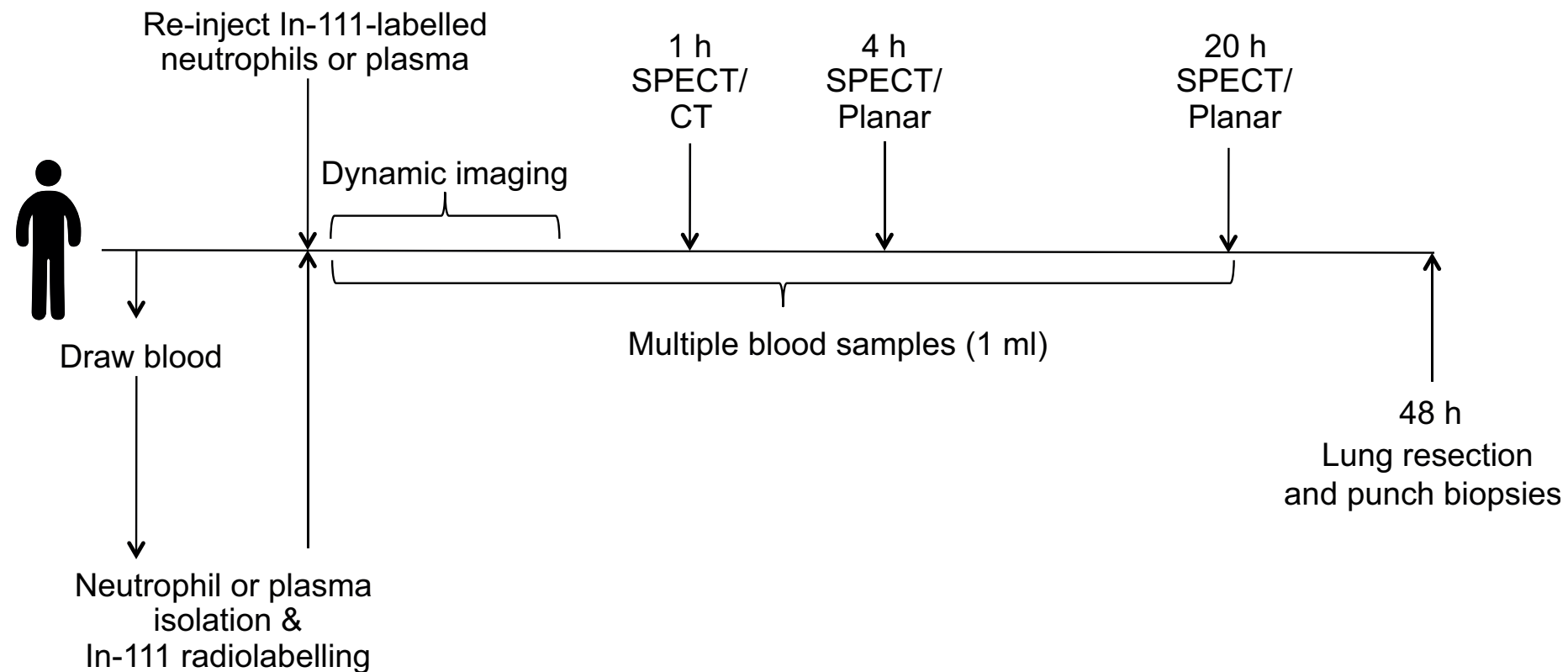

Supplement: Supplementary data [file thoraxjnl-2020-214642supp002.pdf]

Suppl. Figure 2

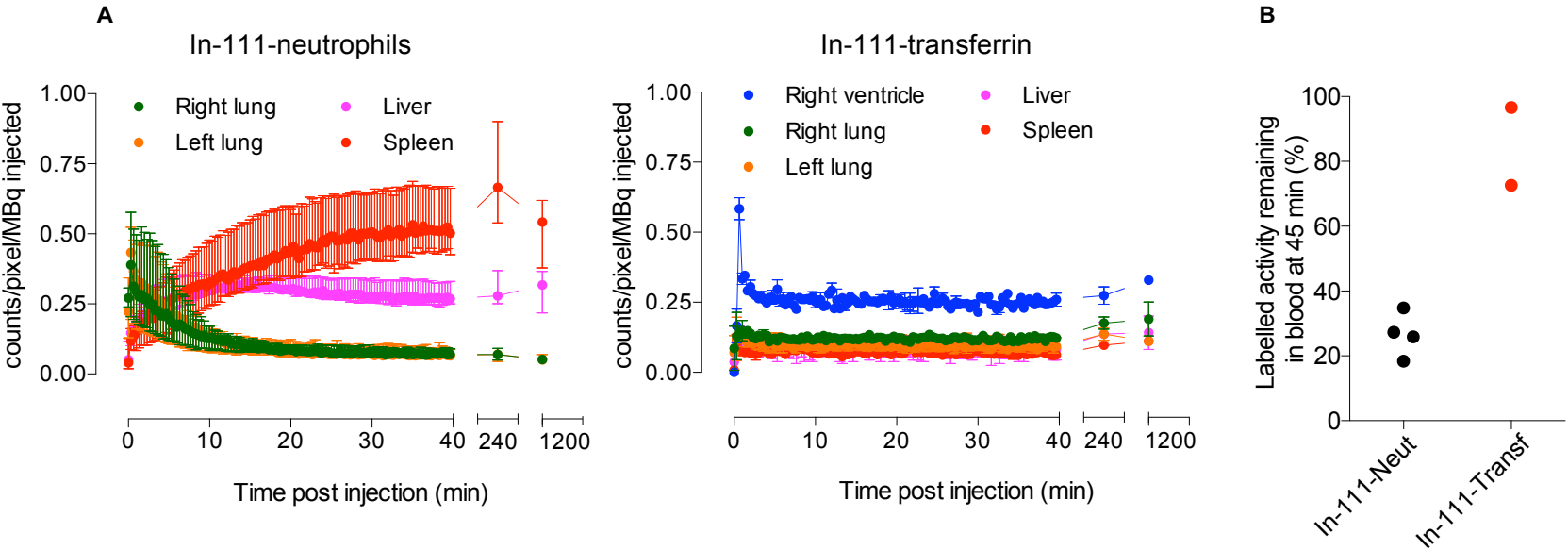

Supplement: Supplementary data [file thoraxjnl-2020-214642supp003.pdf]

Suppl. Figure 3

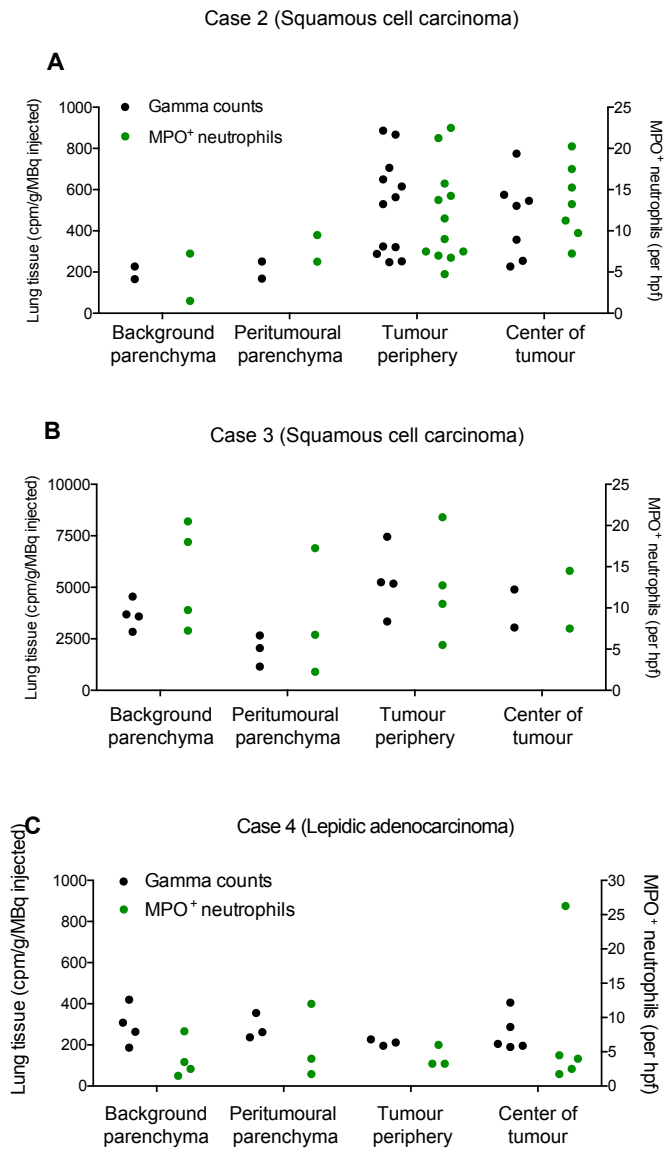

Supplement: Supplementary data [file thoraxjnl-2020-214642supp004.pdf]
